# Diseases of the Ear, Nose and Throat and Their Accessory Cavities

**Published:** 1897-12

**Authors:** 


### Book Notices.

DISEASES OF THE EAR, NOSE AND THROAT AND THEIR ACCESSORY CAVITIES.—A condensed Text-Book. By SETH SCOTT BISHOP, M. D., LL. D., Professor in the Chicago Post-Graduate Medical School and Hospital; Surgeon to the Illinois Charitable Eye and Ear Infirmary; Consulting Surgeon to the Illinois Masonic Orphans' Home and to the Silver Cross Hospital of Joilet; formerly Surgeon to the South-Side Free Dispensary and to the West-Side Free Dispensary; Member of the International Medical Congress, The Pan-American Medical Congress, The American Medical Association, The State Medical Societies of Illinois and Wisconsin, The Chicago Pathological Society, etc. Illustrated with 100 Colored Lithographs and 168 Additional Illustrations. One volume, Royal Octavo, pages xvi–496. Extra Cloth, $4.00 net; Sheep or Half Russia, $5.00, net. The F. A. Davis Co., Publishers, 1914 and 1916 Cherry Street, Philidelphia; 117 W. Forty-Second Street, New York; 9 Lakeside Building, Chicago.

The author states, in his preface to this volume that it was designed, first, to help students in preparing for their degree, second, for those progressive practitioners who wish to acquire the proficiency necessary to properly treat those patients who are unable to visit specialists; and third, for those who are gradually exchanging their general practice for special work in these branches. While it was not intended primarily for specialists, yet it will be valuable to them as a work of ready reference and in bringing information on the subjects treated down to the present date. The subjects are simplified and condensed except in that part of the book which deals with the most recent valuable methods of treatment as the latest developments concerning diphtheria, the blood serum therapy, the medical and surgical management of mastoid diseases, the most successful treatment of hay fever, the improved compressed-air instruments, vaporizing apparatus, inhalents, etc. These subjects are treated in greater detail and are given especial prominence because no books equivalent to this is now available which contains recent information. S. E. H.

